# Rapid Interval Recurrence of Glioblastoma Following Gross Total Resection: A Possible Indication for GammaTileⓇ Brachytherapy

**DOI:** 10.7759/cureus.19496

**Published:** 2021-11-12

**Authors:** Teresa P Easwaran, David Sterling, Clara Ferreira, Lindsey Sloan, Christopher Wilke, Elizabeth Neil, Rena Shah, Clark C Chen, Kathryn E Dusenbery

**Affiliations:** 1 Department of Radiation Oncology, University of Minnesota School of Medicine, Minneapolis, USA; 2 Department of Neurology, University of Minnesota School of Medicine, Minneapolis, USA; 3 Department of Hematology-Oncology, North Memorial Health Cancer Center, Robbinsdale, USA; 4 Department of Neurosurgery, University of Minnesota School of Medicine, Minneapolis, USA

**Keywords:** gamma tile, cesium-131, gross total resection, surgically implanted radiotherapy, radiotherapy, glioblastoma, intraoperative brachytherapy

## Abstract

Glioblastoma recurrence between initial resection and standard-of-care adjuvant chemoradiotherapy (CRT) is a negative prognostic factor in an already highly aggressive disease. Re-resection with GammaTileⓇ(GT Medical Technologies Inc., Tempe, AZ) placement affords expedited adjuvant radiation to mitigate the likelihood of such growth. Here, we report a glioblastoma patient who underwent re-resection and GammaTileⓇ (GT) placement within two months of the initial gross total resection due to regrowth that reached the size of the original presenting tumor. The patient subsequently received concurrent temozolomide and 60 Gy external beam to regions outside of the brachytherapy range, fulfilling the generally accepted Stupp regimen. The patient tolerated the treatment without complication. The dosimetrics and implications of the case presentation are reviewed.

## Introduction

The standard of care for glioblastoma involves maximal safe resection followed by concurrent chemoradiation therapy (CRT) that is initiated between two to six weeks post-surgical resection to afford wound healing [1-2]. Unfortunately, growth of the microscopic, residual tumor adjacent to the resection cavity continues during this wound healing period, leading to MRI-detectable regions of contrast enhancement that can appear as early as a week post the index surgery. Expectedly, detection of such macroscopic regrowth is not uncommon, with reports suggesting that such phenomena occur in more than half of all glioblastoma patients [3,4]. Such regrowth is associated with poor progression-free and overall survival [5]. Significant variability exists in the clinical management of patients with early tumor regrowth, including repeat surgery or immediately proceeding to CRT. If re-resection is an option, there is little agreement in terms of the optimal timing of CRT post-resection to minimize wound breakdown. 

GammaTileⓇ (GT Medical Technologies Inc., Tempe, AZ) is a low-dose rate (LDR) brachytherapy platform that utilizes Cesium-131 (Cs-131) seeds embedded into a resorbable collagen matrix implanted at the time of surgery and is FDA-cleared for use in newly diagnosed malignant intracranial tumors and all recurrent intracranial tumors [6]. In cases of rapid glioblastoma regrowth post-index resection, repeat resection with GammaTileⓇ (GT) placement achieves initiation of antitumor radiotherapy immediately at the time of the procedure, while affording time for wound healing prior to external beam radiation. Here we present a glioblastoma patient who underwent gross total resection (GTR) in an index surgery but the tumor rapidly regrew to the dimensions of the original tumor within two months of surgery. The patient then underwent re-resection with GT placement followed by CRT with external beam radiation therapy (EBRT) directed to the T2/FLAIR region outside of the brachytherapy range four weeks after the repeat surgery. 

## Case presentation

Initial presentation and maximal safe resection with close interval recurrence

**Figure 1:**
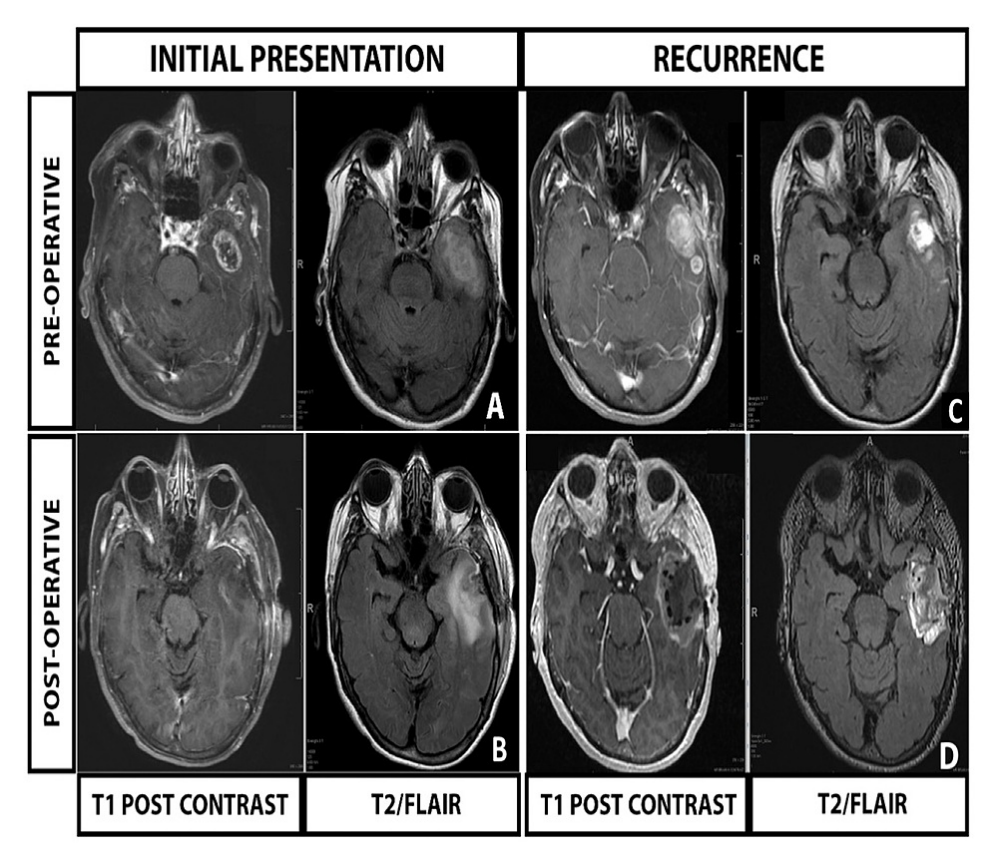
Representative imaging for the patient’s index presentation and recurrence (A) Post-contrast and T2/FLAIR imaging at presentation, (B) Initial post-operative, post-contrast and T2/FLAIR imaging demonstrating maximal safe resection (GTR), (C) Post-contrast and T2/FLAIR imaging at recurrence, and (D) Recurrence post-operative imaging, post-contrast and T2/FLAIR with GT placement.

A 70-year-old woman with a past medical history notable for recurrent pulmonary embolism presented with generalized tonic clonic seizure. Workup included a brain MRI with and without contrast demonstrating a heterogeneously enhancing 2.7 x 1.9 x 2.5 cm in the left temporal fossa with associated surrounding FLAIR (Figure 1A).

The patient underwent gross total resection of the lesion and remained neurologically nonfocal (Figure 1B). Pathology revealed findings consistent with an IDH-wildtype, MGMT promoter methylated glioblastoma. The treatment planning brain MRI three weeks postoperatively demonstrated recurrence of a nodular contrast-enhancing lesion 2.6 x 2.1 x 1.6 cm in the previous resection cavity (Figure 1C).

Re-resection and GT placement

The case was reviewed in a multi-disciplinary conference, with consensus recommendation for presenting the patient with options of initiating CRT or repeat resection with GT placement. After careful discussions, the patient opted to undergo repeat resection with GT placement. The patient underwent an 5-aminolevulinic acid [7] and intra-operative stealth MRI-guided resection to achieve a gross total resection (Figure 1D). During the procedure, six GT tiles (24 seeds) were placed into the resection cavity [8]. Placement of the GT added less than five minutes to the overall procedure. The patient tolerated the procedure without complication and was discharged home on postoperative day one. 

Dosimetry and external beam treatment planning

To determine the number of tiles required, a pre-operative gross tumor volume (GTV) was drawn and a volume in cm2 was calculated using the MRI of the patient at re-presentation. This GTV does not include the skull side surface that would not be included in the treatment volume. The number of tiles to line the surface of the resection cavity was estimated by taking the estimated drawn GTV by the area of the GT surface (4 cm2). Each GT seed had an activity of 3.52U (14.08U per tile), thus bringing the total activity implanted to 84.48U (6 tiles). The prescribed dose was 60 Gy to a margin 0.5 cm deep to the surgical cavity. Dose calculation utilized TG-43 formalism. Planning MRI three weeks following re-resection and GT placement revealed the rim of nodular T1 contrast-enhancement but no grossly notable growth. The T2/FLAIR signal remained unchanged. 

**Figure 2:**
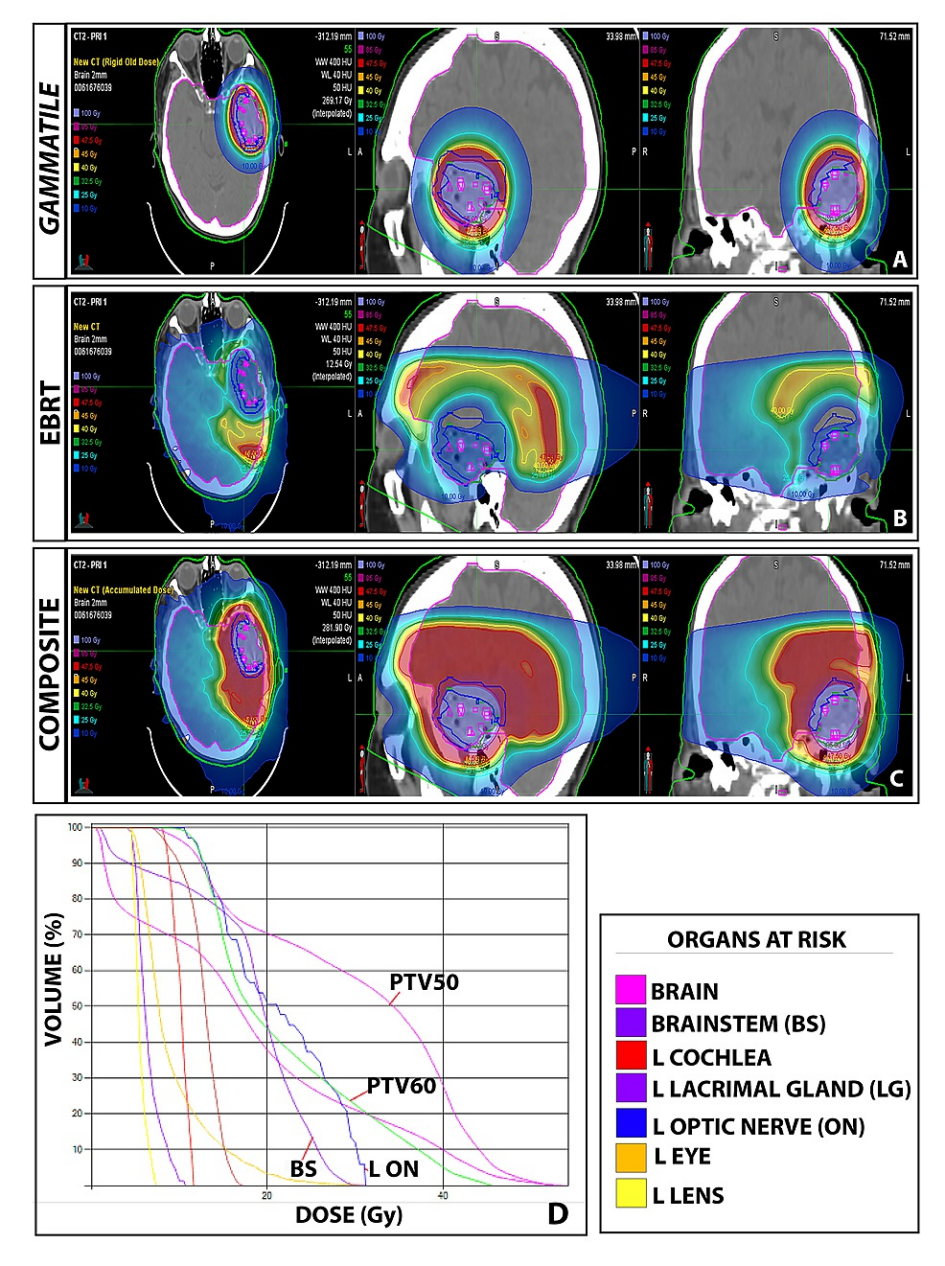
Radiation planning following re-resection and GT placement (A) Dosimetric evaluation of GammaTile, (B) external beam radiotherapy (EBRT), (C) composite, and (D) dose-volume histogram.

In delineating treatment volumes for the GT dose, a residual GTV (GTVr) was created to boost the nodular T1 contrast-enhancing area with a 1 cm radial margin to a cumulative minimum dose of 60 Gy (Figure 2A) as gross disease that had presumably not received the full dose from the implant.

A high-risk clinical treatment volume (HR-CTV) was defined as a 5 mm expansion from the resection cavity wall in addition to GTVr. The HR-CTV received a total dose of 60 Gy. 

As a rim of tissue around the resection cavity 0.5 cm thick had received 60 Gy from the brachytherapy with steep dose fall off, a supplementary external beam plan was then created to give variable doses to the areas at risk using inverse planning (Figure 2B). Taking into account the dose already delivered by GT, concentric dose rings were created to ascribe remaining dose to ultimately bring the total radiation dose to the T2/FLAIR to 50 Gy. At the same time, the plan was designed to boost the nodular T1 contrast-enhancing area with a 1 cm radial margin to a cumulative minimum dose of 60 Gy. To accomplish delivering this complex physical dose distribution, a plan was generated to be treated on a Radixact treatment machine (Accuray Inc., Sunnyvale, CA). The composite dose and dose-volume histogram is shown in Figures 2C-2D, respectively.

Starting four weeks post re-resection/GT, daily oral temozolomide (TMZ) was initiated concurrently with the EBRT plan described above. Her performance status remains excellent. 

## Discussion

Progression between surgery and initiating radiotherapy

For glioblastoma and other central nervous system cancer, it is generally accepted that clinical survival is inversely correlated with the tumor growth rate [9,10]. Because of the fixed volume of the cranial vault, growth of neoplastic tissues necessarily results in the displacement of the normal cerebrum, an effect often referred to as “mass effect”. And, the magnitude of the mass effect is inversely correlated with clinical survival [11]. Additionally, invasive tumor growth into eloquent cerebrum may compromise neurologic function, which negatively affects clinical survival [12]. Supporting these observations, patients afflicted with glioblastoma showing features of rapid growth, as evidenced by macroscopic enlargement of contrast enhancing regions on MRI prior to surgical resection [3] or prior to radiation therapy, showed worsened survival relative to glioblastoma patients without such rapid regrowth. For instance, Palmer et al. reported overall survival of 11.5 months for glioblastoma patients with evidence of macroscopic growth prior to surgery compared to the overall survival 20.1 months without such progression [5]. In another report by Lakomy et al., the median survival for glioblastoma patients with enlargement of contrast enhancing lesions on MRI prior to radiation treatment was 10.7 months. In contrast, the median survival for glioblastoma without such findings was 18.7 months [13]. Similar findings were reported by Pirzkall et al. [4].

The current paradigm for the timing of the Stupp regimen relative to surgical resection is largely based on a meta-analysis of 2855 patients in 16 RTOG trials, where postponement of radiotherapy of 4-6 weeks post surgery was associated with better outcomes than radiotherapy within two weeks after surgery [14]. There are published case series demonstrating worse outcomes, including overall survival and Karnofsky’ Performance Score (KPS), when radiotherapy is initiated after six weeks [15-17]. In this context, the radiotherapy for the presented case was planned for one month post surgical resection, with a treatment planning MRI prior to radiation therapy. The finding of significant tumor growth during this period places the patient in a poor prognostic group. Since there is currently no consensus in the treatment of this patient subset, the case was reviewed in a brain tumor conference. Key discussion points during the conference included: 1) the potential benefit of surgical resection as means of cyto-reduction, though class I data supporting re-resection in glioblastoma remain elusive [18]; 2) the data supporting improved local glioblastoma control by brachytherapy in the face of the absence of class I data in terms of brachytherapy impact on glioblastoma survival [19]; 3) the lack of definitive data for brachytherapy in the context of rapidly progressing glioblastoma; and 4) the merits of proceeding with CRT when knowing the poor prognosis associated with rapidly growing glioblastomas. Ultimately, the recommendation was to offer the patient the option of CRT as well as repeat resection and GT placement. The patient was dismayed with the poor prognosis associated with CRT and opted to pursue repeat resection with GT placement.

In recent years, Cs-131 has emerged as a promising isotope for brachytherapy for CNS tumors [20], and GT was designed based on this literature. The half-life of Cs-131 is significantly shorter than that of Iodine-125 (I-125) (9.7 vs 59.4 days, respectively) with a mean photon energy of 30.4 KeV. This short half-life and lower energy allow for permanent implantation, with an added benefit of having no need for a second procedure to remove the isotope. In the case of GT, four Cs-131 seeds are embedded within an absorbable 2 cm x 2 cm collagen matrix. The modular nature of each tile, their pliability and adherence of the collagen to the resection make implantation streamlined. The collagen offsets the seeds by 3 mm acting as a “spacer” thereby potentially reducing the likelihood of focal necrosis around the sources. This delivers 120-150 Gy at the matrix surface, 60-80 Gy at 5 mm depth, with rapid dose fall off thereafter [8]. 

The patient tolerated the procedure well. The absence of detectable glioblastoma regrowth during the healing period from the repeat surgery contrasts that of the notable tumor growth after the index surgery and suggests efficacy of GT in achieving local control. The patient tolerated subsequent CRT four weeks after repeat resection/GT placement, suggesting that GT can be safely combined with subsequent EBRT and TMZ therapy. During radiation planning, the brachytherapy dose was not converted into equivalence dose of EBT (EqD2), so the EqD2 of this combined regimen probably resulted in a modest dose escalation in areas in close proximity to the resection cavity. The magnitude of this escalation will be better quantified in a planned Phase II clinical trial.

## Conclusions

The management patients who suffer rapid interval recurrence of glioblastoma remains a challenging problem. GT implantation with re-resection affords a possible new treatment strategy with specific advantages. The short half-life of Cs-131 results in 90% of the dose in the first month with the initiation of dose delivery even while the patient is in the operating room. Cs-131 is embedded in absorbable collagen, which acts as a spacer separating the source from direct contact with brain parenchyma, with potentially less risk of radionecrosis. The insertion adds less than five minutes to surgery. The sources are permanent without a need for subsequent removal. 

Herein we report the use of re-resection, GT implantation, and subsequent CRT in a patient with rapid glioblastoma growth after index resection. The approach offers a nuanced balance between the administration of anti-tumor radiotherapy and the need for surgical wound healing. A Phase II trial is planned to determine the impact of this approach on clinical outcomes.
